# Ruptured Valsalva Sinus Aneurysm to Pericardium Simulated Aortic Root Dissection

**Published:** 2014-04-01

**Authors:** Tahereh Davarpasand, Ali Hosseinsabet, Kumars Abassi, Sorya Arzhan

**Affiliations:** 1 Tehran Heart Center, Tehran University of Medical Sciences, Tehran, IR Iran

**Keywords:** Sinus of Valsalva, Dissection, Cardiac Tamponade, Echocardiography

## Abstract

Ruptured valsalva sinus aneurysm to pericardium is a rare condition. Here, we described a case presented with tamponade. Initially, hemopericardium was partially drained and then, imaging evaluations were done. Transesophageal echocardiography showed limited dissection of aortic sinus and CT angiography of the ascending aorta showed deformed dilated right coronary sinus. Besides, surgery showed that windsock tract of the right coronary sinus had ruptured into the pericardium with avulsed right coronary aortic cusp. This case indicated a rare cause of cardiac tamponade and insufficiency of imaging modalities for making an accurate diagnosis.

## 1. Introduction

Sinus of Valsalva Aneurysm (SVA) is defined as a significant dilatation of the aortic wall located between the aortic valve and the sinotubular junction. According to their location relative to the coronary arteries, valsalva sinuses are designated as the Right Coronary Sinus (RCS), Left Coronary Sinus (LCS), and Non-Coronary Sinus (NCS) aneurysm (1). Ruptured SVA is a rare disease that comprises 0.96% of all cardiac surgeries (2).

The causes of ruptured SVA can be congenital or acquired trauma (3), infection (4), or degenerative diseases (4). Moreover, the coexistent usual abnormalities are ventricular septal defect (4-8) and anomalies of the aortic valve (4, 8). RCS is the most frequently affected valsalva sinus followed by NCS and, rarely, LCS (5-8). Furthermore, the most common complication of SVA is rupture of SVA into the right ventricle or atrium and rarely towards the left chambers (4-8). Ruptured SVA may produce serious hemodynamic instability, such as acute heart failure (9) or sudden death (10). Early surgical intervention is the treatment of choice.

Here, we report a rare type of right SVA ruptured to pericardium, resembling to aortic sinus dissection, which was misdiagnosed as “idiopathic pericardial effusion”.

## 2. Case Report

A 43 year old man without cardiovascular risk factors, other chronic diseases, and history of trauma had a history of 20 days of progressive dyspnea on exertion after two episodes of flu-like symptoms, such as fever and chill. He was hospitalized in another center for more evaluation and was discharged with diagnosis of idiopathic moderate pericardial effusion due to viral infection on ibuprofen. He came to our center after 2 days because of exacerbated dyspnea, NYHA III–IV, orthopnea, and palpitation. Physical examinations showed heart rate = 114 bpm, blood pressure = 110 / 60 mmHg, distended jugular veins, and holodiastolic murmur along the left parasternal line without thrill. In addition, the electrocardiogram showed sinus tachycardia, left axis deviation, right bundle branch block, and Q wave in III and aVF leads. Besides, normal lung vascularity and cardiomegaly (round shaped heart) were observed in the chest X-ray. In the first lab data, WBC count (12510 / µL), Hgb (8.9 g / dL), ESR (60 mm / hr), and CRP (0.94 mg / L) were abnormal.

Moreover, Transthorcic Echocardiography (TTE) in the emergency department documented mild left ventricular enlargement with normal ejection fraction (55%), normal right ventricle size, mild right ventricular dysfunction, mild mitral regurgitation, moderate to severe aortic regurgitation, mild tricuspid regurgitation, severe pericardial effusion in favor of tamponade, and normal ascending aorta and aortic arch without visible dissecting flap in the aorta. Therefore, the patient was referred to the operating room for emergent pericardiocentesis. While operation, the surgeon was encountered with hemopericardium and pericardium was full of blood and fresh clots. Thus, he was suspected of accompanied missed aortic dissection with hemopericardium. After some clot removal, the patient was admitted in the ICU for ruling out the aortic dissection.

**Figure 1.**
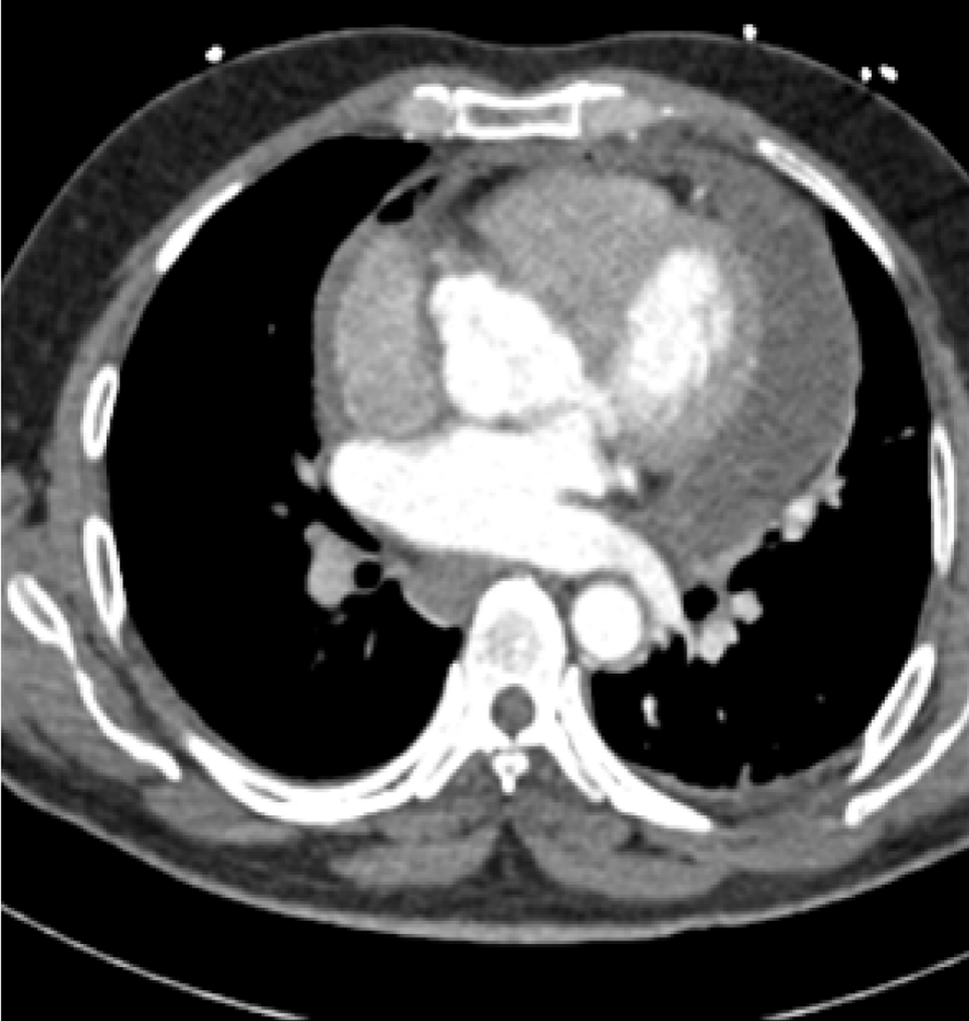
CT Scan Image of the Aortic Sinus that Showed Dilated Distorted RCS

**Figure 2.**
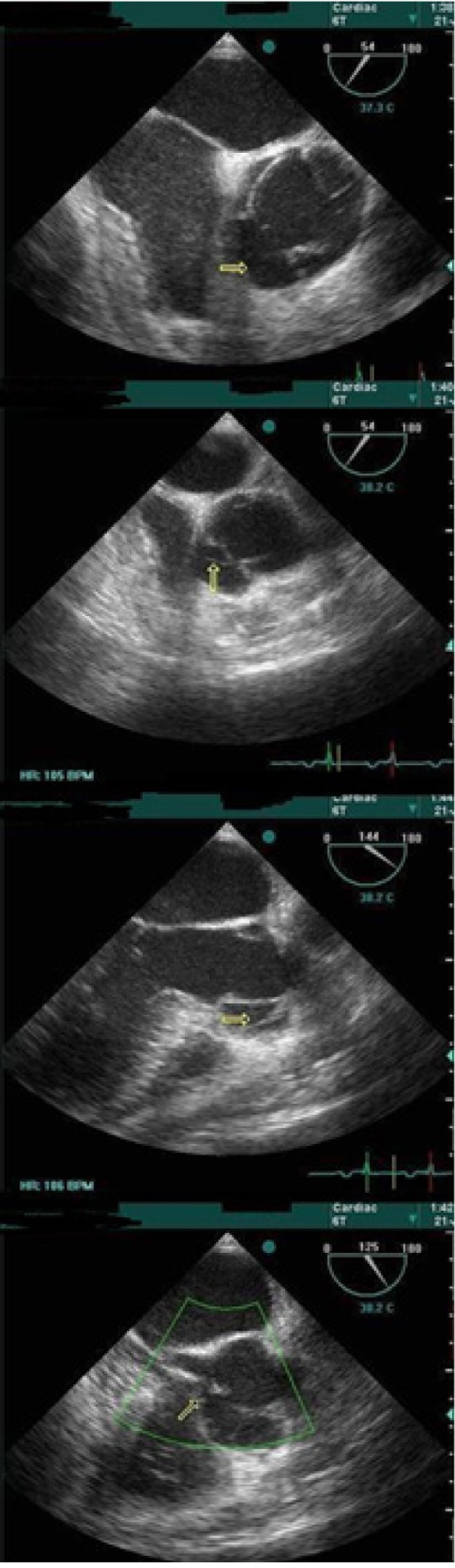
TEE Images First image (top) shows free space in the right coronary-noncoronary commissure. The second image shows the echogenic line as a dissecting flap in cross section. The third image shows the echogenic line as a dissecting flap in long axis. The forth image shows the connection orifice of the aorta and the left ventricle.

After few hours, multislice (256 slices) CT angiography was performed, but aortic dissection or intra mural hematoma was not seen, and ascending, arch, and descending parts of the aorta and coronary arteries were normal. The only abnormal finding was severe pericardial effusion and dilated deformed RCS (Figure 1). Because of high suspicion of the cardiac surgeon to pathology of the aorta, TTE and Transesophageal Echocardiography (TEE) were repeated. Echocardiography findings showed a localized dissection in the right aortic sinus, resulting in false lumen, from which the right coronary artery seemed to originate. The findings also revealed ruptured right coronary-nonocoronary commissure and that the false lumen had perforated to the left ventricular outflow tract with 7mm orifice. This rupture had, in turn, resulted in severe eccentric aortic regurgitation and mild to moderate central aortic regurgitation. Severely thickened, concentrated, and semiliquid pericardial effusion was also visible (Figure 2). 

After echocardiography, the patient was prepared for aortic repair surgery. The procedure was done via median sternotomy with cardiopulmonary bypass. The aorta was cross clamped. A classic windsock tract was seen arising from the RCS protruding into the pericardial sac. However, the other sinuses were normal. The RCS was ruptured into the pericardial space. Additionally, the base of the right coronary cusp avulsed from aortic annulus and the cusp was flittering in the aortic root. The orifice of the right coronary artery was not compromised. Pericardial sac was full of fresh clot. The opening of the aneurysm (2 * 4 mm) was resected by a small windsock and was closed using a pericardial patch from the aortic side and a 0.6 mm Gortex patch from outside. Moreover, the aortic valve was replaced by a mechanical aortic valve because of repair impossibility. Cardiopulmonary bypass time was 130 min and aortic clamping time lasted for 93 min. Sternum was closed immediately after the surgery and the patient had a normal recovery.

## 3. Discussion

The incidence rate of SVA is low accounting for 0.15 - 1.5% of all cardiac surgeries (1). The etiology of SVA can be congenital or acquired (3, 4). Besides, the frequency of ruptured SVA varies according to the location, the most common of which being RCS followed by NCS and LCS (5, 6, 8). Rupture of SVA occurs most frequently at the RV followed by the right atrium, the left ventricle, and rarely the pericardium (5-8). Extracardiac ruptures are rare and more common in acquired SVA. They are usually fatal (due to acute tamponade) and occur towards the pericardium or the pleural space (4, 5). Aortic regurgitation is also present in some cases (5-8). In our case, associated aortic insufficiency seemed to result from the secondary rupture of the right coronary cusp-noncoronary cusp commissure and base of the right coronary cusp from the annulus.

Up to now, several cases of ruptured sinus of valsalva into pericardium have been reported (10-17), three of which being dead (10, 11, 13) and the other three being survived (12, 14, 15, 17). Only one of the survived cases had ruptured RCS into pericardium with tamponade similar to our case, which was probably because of small ruptured orifice and intermittent small leakage resulting in plastic pericarditis. The unique features of our case were long clinical period and that ruptured sinus of valsalva anatomy resembled aortic root dissection in TEE and did not clearly appear in CT angiography.

In general, TTE with color Doppler can correctly but often suboptimally establish the diagnosis of a ruptured aneurysm in 75% of the patients, thereby resulting in imprecise delineation of the anatomy and anatomic relations of the aneurysm and its associated lesions. These limitations were obviously present in our case, in which TTE was normal, except for the unknown origin of severe aortic regurgitation and severe pericardial effusion. To date, TEE has become the gold standard for diagnosis of this lesion. Associated with color Doppler, TEE precisely defines the location, morphology, size, associated lesions, and complications of the defect (16). In our case, however, SVA was ruptured to pericardium and sealed off by thrombosis and, consequently, no shunt was detected by color flow Doppler study. In echocardiography also, windsock appeared as an echofree space around the aortic sinus that was separated from the main aortic tunnel, falsely resembling the limited dissection of the aortic root with false lumen with ruptured aortic annulus, resulting in aortic regurgitation. Of course, some of these findings were true and 3D echocardiography could help us make the correct diagnosis (18). Nowadays, development of a new generation of multi slice CT scan has created a range of opportunities in the field of CT angiography (19), but it was not helpful in our case. When congenital abnormalities of the thoracic aorta are suspected, confirmation of this abnormality can be made by MRI, allowing precise evaluation of the aortic root and thoracic aorta (20).

In conclusion, hemopericardium can be one of the presentations of ruptured sinus valsalva into pericardium. In these cases, imaging modalities have some limitations and accelerated surgery should be considered.
